# Comprehensive Analysis of Current Treatment Approaches for Keloids in Pediatrics: A Systematic Review

**DOI:** 10.7759/cureus.50290

**Published:** 2023-12-10

**Authors:** Reem A Al Zahrani, Wejdan N Alotaibi, Zainab M Almanasef, Ibtihal Malawi, Lujain A Mohammed, Rana A Algahamdi, Abdulaziz A Almohanna, Ahmed N AlKhaytan, Rahaf J Albishi, Yazeed A Alsofyani, Fahad K Aljindan

**Affiliations:** 1 Medicine and Surgery, Umm Al Qura University, Makkah, SAU; 2 Pediatrics, Armed Forces Hospital Southern Region, Khamis Mashait, SAU; 3 College of Medicine, Royal College of Surgeons in Ireland, Dublin, IRL; 4 Dermatology, King Fahad General Hospital, Makkah, SAU; 5 College of Medicine, King Khaled University, Abha, SAU; 6 College of Medicine, Al Bahah University, Al Bahah, SAU; 7 College of Medicine, Imam Mohammad Ibn Saud Islamic University, Riyadh, SAU; 8 College of Medicine, Qassim University, Buraydah, SAU; 9 College of Medicine, King Abdulaziz University, Jeddah, SAU; 10 College of Medicine, Taif University, Taif, SAU; 11 Plastic Surgery, King Abdullah Medical City, Makkah, SAU

**Keywords:** children, pediatric, keloid, therapy, intervention, management, treatment

## Abstract

Keloids, benign fibrous growths resulting from atypical skin responses to injuries, present a complex challenge in dermatology. These lesions, characterized by excessive collagen production, often lead to physical discomfort and psychological distress. While various treatment methods exist, the lack of a universally effective modality underscores the need for a systematic evaluation of current approaches.

This systematic review aims to comprehensively analyze the current available treatment modalities used for the management of keloids in the pediatric population in terms of their effectiveness, safety, and quality of life outcomes.

The review adheres to the Preferred Reporting Items for Systematic Reviews and Meta-Analyses (PRISMA) guidelines. A comprehensive search was conducted on PubMed and Google Scholar databases to identify relevant studies published in English. The review specifically focused on randomized controlled trials involving patients under 18 diagnosed with keloids, assessing different treatment modalities, and reporting validated measures of treatment efficacy, safety outcomes, and quality of life. The risk of bias was assessed using Cochrane’s Risk of Bias Tool for randomized studies to ensure the methodological quality of the included trials.

Four studies met the inclusion criteria, collectively involving 196 pediatric patients. Treatment interventions included glucocorticosteroid and fusidic acid cream with silicone gel patches, botulinum toxin type A injections, and Scarban silicone gel sheets. Patient-reported outcomes exhibited varying degrees of improvement in scar size, vascularity, and pliability. Complications, such as rash and wound infection, were reported in some cases.

Based on our review of the selected studies and due to the incompletely understood pathogenesis of keloids, there is an ongoing lack of universally effective treatment modality for the management of keloids resulting in their persistently high recurrence rate.

## Introduction and background

Keloids, characterized as benign fibroproliferative dermal tumors, result from aberrant wound healing responses, predominantly instigated by trauma, infections, and additional contributing factors including repeated skin stress, hormonal fluctuations, and genetic predisposition, demonstrating a higher prevalence among populations of African, Asian, or Hispanic ancestry [1]. These fibrous tumors appear as dense masses within and beyond the boundaries of a wound, after injury to the skin [2,3]. The lesion’s distribution is equal between both sexes and its incidence peaks in the second and third decades of life [4]. In addition to the aesthetic issues they cause, keloids can also be pruritic, painful, and psychologically draining to patients [5].

Many parts of the pathogenesis of keloids still remain largely unknown, but it is agreed upon that they occur when there is an imbalance in the healing process, causing fibroblasts to excessively produce collagen at a rate and amount much higher than that produced normally [5]. The prevalence of keloids is relatively high, as almost 11% of the 100 million scars that develop yearly progress into becoming keloids [6]. The rate of keloid formation varies depending on race; Blacks, Hispanics, and Asians have been reported to have a higher incidence of keloids than other racial groups. Generally, Whites are 15% less likely to develop keloids when compared to dark-skinned populations [7]. In addition to the aforementioned predisposing component, it has been noted that a positive family history can be a very significant risk factor for individuals; some studies also suggest an autosomal dominant mode of inheritance with variable expression and penetrance [8]. In fact it is estimated that 5-10% of cases have familial keloids [6].

Keloids still remain an enigma to dermatologists and plastic surgeons, as there is no clear basis on which a clear management plan can be conducted [9]. While various treatments, such as surgical excision, intralesional steroids, oral antihistamines, cryotherapy, laser removal, and immunotherapy, are employed, their recurrent nature persists as a significant issue [5]. Presently, no current single modality of treatment has been validated to universally manage keloids, without a high recurrence rate [10]. Often, multiple modalities are used at once, as recent studies have shown that combination therapies are superior to monotherapy in reducing the likelihood of recurrence [10].

Currently, there are still many unexplained facets about the pathogenesis and treatment of keloids, and this scarcity of knowledge becomes especially relevant when exploring the issue amongst the pediatric population. There are many studies done previously assessing single-treatment modalities on pediatric patients, but no study investigated the problem on a wider scope. This presents a considerable dilemma, as we see many managements all used just as commonly, without a clear sight of which is more efficacious. With regard to the extensive amount of treatment options available for keloids, this review aims to comprehensively analyze treatments of keloids in pediatric patients, including the effectiveness, safety, and quality of life outcomes associated with different treatment modalities. In doing so, we expect to assist physicians in selecting the most appropriate approaches to treating keloids, aiding in clinical decision-making.

## Review

Methods and materials

Registration

This comprehensive evaluation adhered to the criteria specified by the Preferred Reporting Items for Systematic Reviews and Meta-Analyses (PRISMA) [11], and utilized Cochrane review methodologies. The study protocol was proactively recorded in the National Institute of Health Research's Prospective Register of Systematic Reviews, PROSPERO [12], (CRD42023441196).

Search Strategies

A comprehensive systematic search was conducted on PubMed and Google Scholar databases, with a focus on studies published exclusively in English and without any geographical or time restrictions. The search strategy was designed through the integration of Medical Subject Headings (MeSH) terms, free text, and Boolean logic operators, following discussions with the senior authors. The selected keywords included: "(treatment OR management OR intervention OR therapy) AND (Keloid) AND (Pediatric OR Children)". Furthermore, a meticulous screening of the references within the included articles was performed to ensure their relevancy to the research topic.

Screening and Selection of Studies

After the database search, all study data were collected and organized in an Excel worksheet. The screening of titles and abstracts to identify relevant articles was performed using the Rayyan software [13], by four independent researchers (WN, ZM, IA, LA). In cases of any inconsistencies, the paper underwent a comprehensive full-text assessment. Subsequently, the full texts of the shortlisted studies were subjected to an eligibility screening by the same four researchers. Any discrepancies were resolved through collaborative discussions, and the final decision on inclusion or exclusion was made after consultation with the lead researcher (RA).

A standardized extraction template was employed to extract relevant data from the full-text articles. The extracted data encompassed key aspects such as study design, sample size, patient characteristics, intervention details, and reported outcomes. Any arising disparities were addressed through consensus among the researchers or referred to the lead researcher (RA) for resolution.

The following data were extracted from each study: first author; year of publication; study design; sample size; cause of lesion; location of lesion; intervention; follow-up duration; outcome measures; reported complication.

The primary outcome of this systematic review was to evaluate the efficacy and prevention potential of various keloid treatments in pediatric patients. Through a rigorous examination of the selected studies, we assessed the effectiveness of treatment interventions for keloids, focusing on keloid size reduction and the prevention of recurrence. As a secondary outcome, we aimed to assess the impact of the identified treatment approaches on the quality of life outcomes in pediatric patients with keloids.

Study Design and Criteria

In our systematic review, we focused on all primary human studies assessing treatment approaches for keloids in pediatric patients. The inclusion and exclusion criteria were as follows.

Inclusion criteria:We considered studies involving patients under the age of 18 who had been diagnosed with keloids. Our scope encompassed all types of keloid treatments, including surgical excision, intralesional corticosteroids injection, laser therapy, radiation therapy, cryotherapy, silicone gel sheets, and other topical treatments. Furthermore, we sought studies with a comparison between these keloid treatments and either a placebo or no intervention. We required studies to report on validated measures of keloid size, safety outcomes, and quality of life outcomes, such as the Vancouver Scar Scale (VSS), adverse events, and the Children Dermatology’s Life Quality Index. Only randomized controlled trials (RCTs) were considered, and studies had to be published in the English language.

Exclusion criteria:Studies that did not meet the inclusion criteria were excluded from our review. We also excluded studies that did not provide outcome data on keloid size, safety outcomes, or quality of life outcomes. Furthermore, studies involving non-pediatric patients and those published in languages other than English were not considered in our analysis.

Risk of Bias and Quality Assessment

The Cochrane Risk of Bias (RoB2) tool [14] was employed for the appraisal of RCTs' quality. This instrument incorporates multiple domains, and the evaluations within each are compiled for an overarching RoB2 judgment across five primary domains. These domains, which are predefined, revolve around trial design, execution, and reporting facets, using a set of 'signaling questions' to extract bias-related data. The assessment then undergoes an algorithmic judgment process, and the outcomes can be categorized as 'low' (if all domains present a low risk of bias), 'some concerns' (if at least one domain presents some level of concern), or 'high' (if at least one domain exhibits a high risk or multiple domains exhibit some concerns).

Data Synthesis 

As the included studies exhibited substantial clinical heterogeneity in their design, interventions, and outcome measures, conducting a quantitative meta-analysis was not feasible. Therefore, the findings were synthesized narratively to provide a qualitative analysis of the data. The narrative synthesis allowed for a comprehensive and descriptive exploration of the diverse treatment approaches for keloids in pediatric patients. Each study's individual characteristics, findings, and limitations were carefully considered, and the results were presented in a coherent and interpretive manner.

Results

Primary Results of the Search

**Figure 1:**
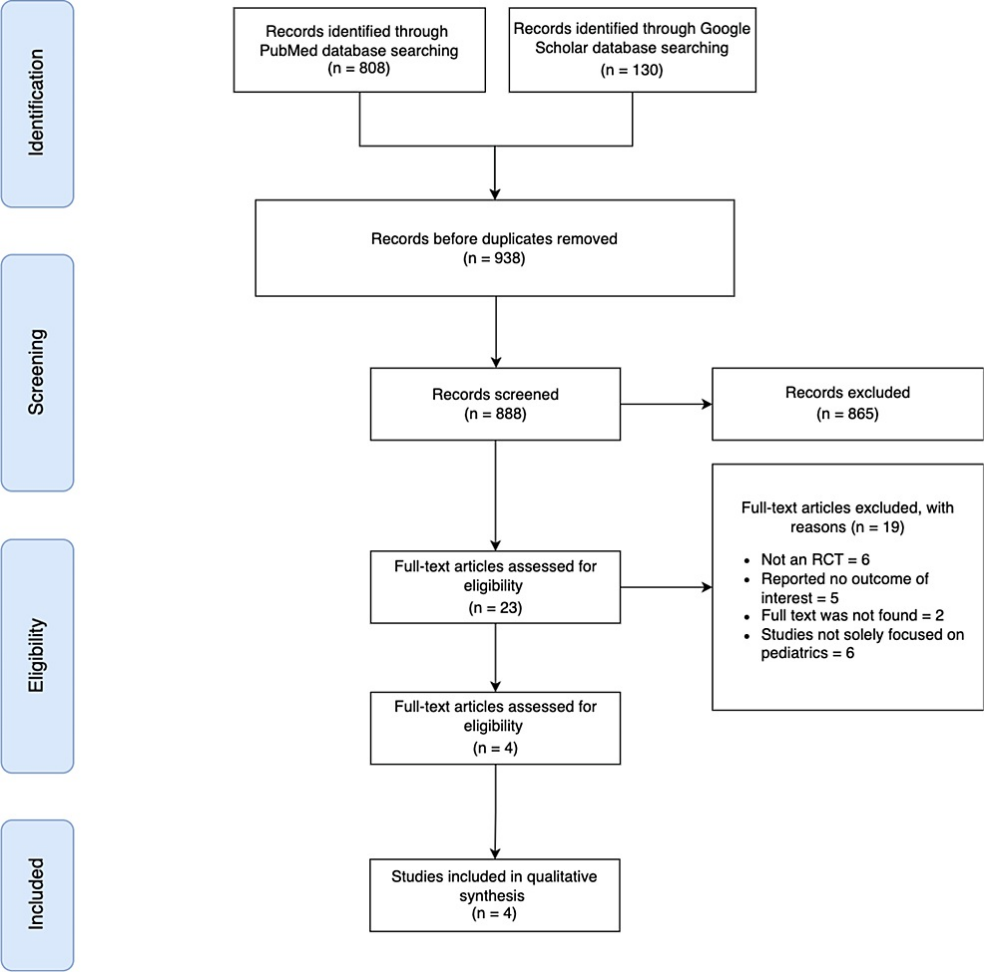
An overview of the studies selection based on Preferred Reporting Items for Systematic Reviews and Meta-Analyses (PRISMA). RCT: Randomized Controlled Trial

Our initial search of two main databases, PubMed and Google Scholar, yielded a total of 938 papers. After removing 50 duplicates (see PRISMA [11] flowchart, Figure 1), we screened the remaining 888 papers by reading titles and abstracts. Subsequently, we excluded 865 papers and retrieved full text for 23 papers to assess their eligibility. Ultimately, only four articles met all the necessary criteria and were incorporated into this systematic review. These included studies encompassed a range of designs, such as double-blinded placebo-controlled randomized clinical trial, randomized intra-patient comparative study, randomized controlled trial, and a prospective, double-blinded, randomized, split-scar study. These studies were published between 2013 and 2023 and originated from various countries, namely Denmark, Egypt, The Netherlands, and Thailand (Table 1).

**Table 1:** Characteristics of the studies included. RCT: Randomized Controlled Trial **** indicates missing information in the corresponding paper.

Paper	Study Design	Country	Population	Gender (M/F)	Sample Size		Ethnicity	Follow-up Duration
Nissen et al., (2022) [15].	Double-blinded placebo RCT.	Denmark	Pediatrics	53/37	115		Whites, Black, Skin Type 2, Skin Type 5.	1 year
									Tawfik et. al., (2023) [16].	Randomized intra-patient comparative study.	Egypt	Pediatrics	9/6	15		Egyptian.	6 months
Katja et al., (2015) [17].	RCT.	The Netherlands	Pediatrics	22/14	36		Caucasians.	1 year
Wananukul et al., (2013) [18].	Prospective, double-blinded, randomized, split-scar study.	Thailand	Pediatrics	17/13	30		****	6 months

*Patient Profiles and Characteristics * 

In this systematic review, a total of 196 patients were included. Among them, 139 patients underwent treatments involving glu-corticosteroid and fusidic acid, injection of botulinum toxin type A, or Scarban silicone gel sheet application. The remaining individuals formed the comparison group without undergoing any treatment. 

The age of participants ranged from 0-17 years, with mean age of 8.79 years. Concerning the distribution of gender in the studies, males constituted a larger group with approximately 59.06% (n=101), while females comprised a total of 40.94% (n=70). Most of the studies included ethnicity information for their participants. The reported ethnicities comprised White (n=66), Black (n=1), Egyptian (n=15), and Caucasians (n=26). However, it's worth mentioning that one study did not provide information regarding the ethnicity of its participants. The follow-up periods in the studies varied, spanning approximately two months to one year. 

The keloids analyzed in these studies were predominantly a result of surgeries performed in the anterior thoracic region. In contrast, the second study focused on keloids that had originated from burns affecting diverse areas such as the trunk, extremities, ears, neck, and shoulders (Table 2).

**Table 2:** Intervention and reported outcomes. **** indicates missing information in the corresponding paper.

Paper	Type of Intervention	Location of Lesion	Cause of Lesion	Outcome	Complication
Nissen et al., (2022) [15].	Cream with glu-corticosteroid and fusidic acid + Silicon gel patches.	Anterior thoracic region	Surgery	Silicone gel sheet ± glucocorticosteroid application during sheet occlusion does not clinically improve scar outcome after central venous catheter removal in children treated for neoplastic diseases.	****
						Tawfik et. al., (2023) [16].	Injection of 5 IU/cm2 of botulinum toxin type A using Neuronox.	Trunk, extremities, ears, neck, and shoulders.	Burn	Marked enhancement in vascularity, pliability, and height of Neuronox-injected lesions after each session, but no change in pigmentation.	No complications were reported
Katja et al., (2015) [17].	Scarban silicone gel sheet "light" (ScarPro NV, Deinze, Belgium).	Anterior thoracic region	Surgery	At 1-year follow-up, the 2-month application group showed significantly smaller scars compared with the group receiving silicone gel sheet treatment for 6 months, but not when compared with the control group.	Wound Infection (1 child), Rash (4 children)
Wananukul et al., (2013) [18].	10% onion extract in silicon derivative (Cybele Scagel, Bangkok Botanica, Bangkok, Thailand).	Anterior thoracic region	Surgery	Scar assessment revealed: 20% of patients achieved complete scar resolution, 30% experienced hypertrophic scarring, and 50% developed keloids.	Pustule (1 child)

The intervention group in the first study [15], consisted of 48.92% (n=68) of participants, with 34 of them receiving cream with glu-corticosteroid and fusidic acid, while the remaining participants received placebo cream with fusidic acid. Both groups received silicon gel patches after central venous catheter removal. In the second study [16], 10.79% of participants (n=15) were included. Each lesion was randomly divided into two parts. One part received intralesional injection of 5 IU/cm2 of botulinum toxin type A every month for six months, using Neuronox (100 U vacuum-dried powder in a single-use vial for reconstitution diluted in 2 ml of sterile, preservative-free 0.9% saline to constitute a solution at a concentration of 5 U/0.1 ml). The other part of the lesion served as the control group.

In the third study [17], a three-arm randomized controlled trial was conducted. Both intervention groups consisted of 18.71% of participants (n=26), who received Scarban silicone gel sheet "light" (ScarPro NV, Deinze, Belgium). The application of the sheet started with a three-hour duration daily and gradually extended by one hour each day until reaching a duration of 20-23 hours. One intervention group (n=12) used the sheet for a period of two months, while the other group (n=14) applied it for six months. The third group (n=10) served as the control group in the study.

In the fourth study [18], where 21.58% of participants (n=30) had their wounds divided into upper and lower parts. Each part of the wound was randomized and assigned to be applied with either 10% onion extract in silicon derivative (Cybele Scagel, Bangkok Botanica, Bangkok, Thailand) or a placebo. The placebo gel comprised water, acrylate, C10-30 alkyl acrylate crosspolymer, polysorbate 20, and fragrance, resembling the color and consistency of the active gel. Topical application was administered twice daily, starting from day seven after open heart surgery and continued for six months.

Patient-Reported Outcomes and Complications

In this section, we present the findings regarding patient-reported outcomes and the occurrence of complications associated with the treatment. The outcomes reported here do not include patients who withdrew from the studies (n=48).

The first study [15], an assessment at 12 months, demonstrated a significant reduction in Vancouver Scar Scale and scar size (0.1 cm) in the intervention group compared to the control group. However, no benefit of glucocorticosteroid was observed. The Patient and Observer Scar Assessment Scale indicated no significant difference between the intervention and control groups. The second study [16], showcased remarkable clinical and statistical improvement in vascularity, pliability, and height of neuronox-injected lesions. The Skin analysis camera system (Antera 3D; Miravex, Dublin, Ireland) confirmed significant changes in vascularity and height. Notably, no correlations were found between Vancouver score improvement and variables such as age, sex, skin type, duration, and lesion type.

In the third study [17], there seems to be a small benefit for scar width with application for two months. However, for hypertrophy, the scar outcome shows no significant difference between the control group and the two-month and six-month treatment groups. In the fourth study [18], onion extract in silicone derivative gel can significantly decrease the incidence of hypertrophic scars from median sternotomy wounds in pediatric patients. Vancouver's score was not statistically significantly different in all visits. 

Complications were observed in some of the studies. In one study, four patients developed a rash, and an additional patient experienced a wound infection due to the use of the Scarban silicone gel sheet [17]. Another study reported a single case of a pustule formation on the area where onion extract in silicon derivative was applied during the sixth month of treatment [18].

Risk of Bias Assessment 

Two reviewers simultaneously and independently assessed the risk of bias for eligible RCTs using the Cochrane RoB2 tool [14]. The Revised Cochrane tool (Table 3) was utilized for this purpose, and all four included RCTs were considered to have a low risk of bias.

**Table 3:** Review authors' judgments about each risk of bias (RoB) item for each included study.

Paper	Bias arising from the randomization process	Bias due to deviations from intended interventions	Bias due to missing outcome data	Bias in measurement of the outcome	Bias in selection of the reported result	Overall RoB
Katja et al. (2015) [17].	Low	some concerns	Low	Low	Low	Low
Tawfik et al. (2023) [16].	Low	Low	Low	some concerns	Low	Low
Wananukul et al. (2013) [18].	Low	Low	Low	Low	some concerns	Low
Nissen et al. (2022) [15].	Low	Low	Low	Low	some concerns	Low

Discussion 

In this systematic review, we conducted a comprehensive analysis of various current treatment approaches for keloids in pediatric patients. Our review aimed to assess the effectiveness, safety, and quality of life outcomes associated with different treatment modalities. The findings from the selected studies shed light on the challenges and complexities of managing keloids in this specific population. Despite the diversity of treatment approaches, our results highlight the ongoing lack of a universally effective modality for keloid management, contributing to the persistently high recurrence rate.

Our research results align with the broader evidence in the field, which acknowledges the difficulty in achieving long-term resolution for keloids. Keloids, characterized by benign fibrous growths resulting from an atypical skin response to injuries, remain enigmatic due to the complex pathogenesis involving fibroblast proliferation and collagen deposition during the healing process. The pathogenesis of keloids remains incompletely understood, leading to their recurrent and treatment-resistant nature.

Limitations of the Evidence

The inclusion of a small number of studies and variations in study designs, interventions, and outcome measures are limitations inherent to our analysis. These variations, however, provide valuable insights into different aspects of keloid management. It is important to critically evaluate the limitations of each study to ensure a comprehensive understanding of their findings.

The study by Nissen et al. focused on cream with glu-corticosteroid and fusidic acid, along with silicon gel patches. While the study demonstrated significant reduction in scar size and Vancouver Scar Scale, it is important to note that the benefit from glucocorticosteroid application, while statistically significant, might not be considered clinically significant [15]. However, recent research suggests silicone gel or sheeting combined with corticosteroid injections as the preferred first-line treatment for keloids [19].

Tawfik et al. conducted an intra-patient comparative study using botulinum toxin type A. Despite marked improvement in vascularity and pliability, the short follow-up duration of 6 months raises concerns about the durability of the observed effects [16]. Additionally, following a seven-month follow-up period, emerging evidence suggests that BXT-A may be comparable to triamcinolone in inducing reductions in keloid scar volume, height, and redness [20].

Katja et al. employed a silicone gel sheet treatment approach, revealing smaller scars in the two-month application group. However, the lack of significant difference in hypertrophy outcomes raises uncertainty about the optimal duration of treatment [17].

Wananukul et al. examined the use of onion extract in silicon derivative for scar resolution. While some patients achieved complete scar resolution, a notable percentage developed keloids, suggesting variability in treatment responses [18].

Limitations of the Review Process

While our review adhered to rigorous methodologies following the PRISMA guidelines, potential biases and limitations in the review processes should be acknowledged. The limited number of included studies and potential publication bias may impact the generalizability of our findings.

Our findings underscore the need for continued research efforts to identify effective and sustainable treatment approaches for pediatric keloids. While the results of the analyzed studies offer valuable insights, it is crucial to consider their limitations. These limitations include small sample sizes, reliance on self-reported data, limited generalizability, short follow-up periods, and potential biases. Acknowledging these limitations enhances the understanding and interpretation of the evidence provided by these studies.

In fact, the absence of a single reliable treatment technique that guarantees a response to therapy and prevents keloid recurrence necessitates further investigation. Prospective therapies encompass a range of topical and injectable medications, surgical procedures, and light therapies. However, the lack of uniform controls in evidence for interventions and outcome variability complicates result evaluation.

Furthermore, future research should consider the cost-effectiveness of keloid therapy in healthcare planning. The subjective nature of assessment tools employed in some studies, such as the Visual Analog Scale (VAS) and VSS, introduces potential bias. The majority of studies were conducted in Europe, primarily involving patients of European ethnicity. Therefore, our findings may not fully represent patients of other ethnicities, particularly individuals of African origin who are more prone to keloid [21].

Despite challenges in comparing results due to heterogeneity in methodologies, patterns began to emerge. We observed a higher representation of men in the patient groups across studies, although keloids are generally believed to affect both genders equally. The high female-to-male ratio in pediatric studies may be influenced by social factors, such as gender-specific practices like ear piercing, which is more prevalent among females and has been associated with keloid earlobe formation [8]. This correlation prompts the need for additional investigation into the potential societal influences contributing to this ratio.

In conclusion, our systematic review provides valuable insights into the treatment approaches for keloids in pediatric patients. While the limitations of the included studies should be acknowledged, our findings contribute to the understanding of keloid management and emphasize the need for future research to address the complexities and variations in treatment outcomes.

## Conclusions

In this systematic review, we comprehensively analyzed the current treatment plans for keloids in pediatric patients as reported in the medical literature. Our evaluation specifically focused on assessing treatment efficacy, safety profiles, and impact on patients' quality of life across various therapeutic approaches. The synthesis of these studies underscores the absence of a universally acclaimed gold-standard treatment strategy for keloid management in this demographic.

Notwithstanding the relatively limited sample, this study offers valuable insights into different aspects of keloid management. The identification of gaps and disparities within the current body of research accentuates the critical necessity for further investigations. These future studies should delve into the intricacies of treatment outcomes, accounting for variations and complexities, as well as considering broader ethnic and demographic factors.

In conclusion, this systematic review contributes a holistic understanding of the prevailing treatment options for pediatric keloids. The absence of a definitive treatment protocol highlights the ongoing challenge in managing this condition. As we navigate these uncertainties, our findings underscore the imperative for continued research endeavors aimed at unraveling the complexities associated with keloid management and refining therapeutic strategies.
